# Roles and Mechanisms of DNA Methylation in Vascular Aging and Related Diseases

**DOI:** 10.3389/fcell.2021.699374

**Published:** 2021-06-28

**Authors:** Hui Xu, Shuang Li, You-Shuo Liu

**Affiliations:** ^1^Department of Geriatrics, The Second Xiangya Hospital, Central South University, Changsha, China; ^2^Institute of Aging and Age-Related Disease Research, Central South University, Changsha, China

**Keywords:** DNA methylation, aging, vascular diseases, endothelial cells, vascular smooth muscle cells

## Abstract

Vascular aging is a pivotal risk factor promoting vascular dysfunction, the development and progression of vascular aging-related diseases. The structure and function of endothelial cells (ECs), vascular smooth muscle cells (VSMCs), fibroblasts, and macrophages are disrupted during the aging process, causing vascular cell senescence as well as vascular dysfunction. DNA methylation, an epigenetic mechanism, involves the alteration of gene transcription without changing the DNA sequence. It is a dynamically reversible process modulated by methyltransferases and demethyltransferases. Emerging evidence reveals that DNA methylation is implicated in the vascular aging process and plays a central role in regulating vascular aging-related diseases. In this review, we seek to clarify the mechanisms of DNA methylation in modulating ECs, VSMCs, fibroblasts, and macrophages functions and primarily focus on the connection between DNA methylation and vascular aging-related diseases. Therefore, we represent many vascular aging-related genes which are modulated by DNA methylation. Besides, we concentrate on the potential clinical application of DNA methylation to serve as a reliable diagnostic tool and DNA methylation-based therapeutic drugs for vascular aging-related diseases.

## Introduction

Vascular aging is characterized by gradual changes in the vasculature structure and function (Laina et al., 2018; Ding et al., 2020). With aging, the structure and mechanical properties of vascular wall alter, i.e., lumen dilation, wall thickening, decreased arterial compliance, and increased arterial stiffness (North and Sinclair, 2012). The anatomical structure of vascular includes intima, media, and adventitia. Significant changes occur in the intima and media in the vascular aging progression (Lakatta and Levy, 2003a). Vascular intima primarily comprises endothelial cells (ECs), media is composed of vascular smooth muscle cells (VSMCs), and vascular adventitia is primarily composed of fibroblasts. Vascular cell senescence triggers cell morphological and functional changes, hence, ECs dysfunction, phenotypic transition of VSMCs, macrophage polarization, and fibroblast differentiation to myofibroblast (Chi et al., 2019). Age is an independent risk factor for vascular disorders (Morgan et al., 2018). To date, the aging population is significantly increasing, and research estimated that by 2040, 22% of people will be over the age of 65 (Heidenreich et al., 2011). Several lines of studies indicated that vascular aging enhanced the incidence and mortality of atherosclerosis (Mahmood et al., 2014), Alzheimer’s disease (AD) (Lakatta and Levy, 2003b), stroke (Lakatta and Levy, 2003a), etc. Vascular aging-related diseases are the leading causes of death among the elderly. Thus, there is an urgent need to identify reliable and efficient diagnosis and treatment for vascular aging-related diseases.

DNA methylation is an epigenetic mechanism involving multiple biological processes such as aging, metabolism, and autoimmune (Jones, 2012). Scholars believe that epigenetics is based on alterations in gene expression levels and does not involve DNA sequence changes (Brunet and Berger, 2014). DNA methylation is a dynamically reversible process regulated by methyltransferases and demethyltransferases and it regulates gene expression by recruiting proteins implicated in gene repression or inhibiting the binding of transcription factors to DNA (Moore et al., 2013). DNA methylation is tightly associated with vascular aging and related disorders. Although studies on the link between DNA methylation and vascular disease have got much attention, the underlying mechanisms and roles of DNA methylation in vascular aging are still not well elucidated.

Since the prevalence and mortality of vascular disorders are closely related to vascular aging, the diagnosis and treatment of vascular aging and related diseases have received significant research attention. Therefore, this review summarizes the current research and recent advances on DNA methylation in vascular aging, revealing the involvement of DNA methylation in ECs, VSMCs, fibroblasts, and macrophages functions. We review the physiological and pathological processes involving DNA methylation in vascular aging-related diseases and represent many vascular disease-related genes that are regulated by DNA methylation. Additionally, we concentrate primarily on the clinical prospect of DNA methylation as an early diagnostic tool and potential DNA methylation-based therapies for vascular aging-related diseases.

## DNA Methylation

**FIGURE 1:**
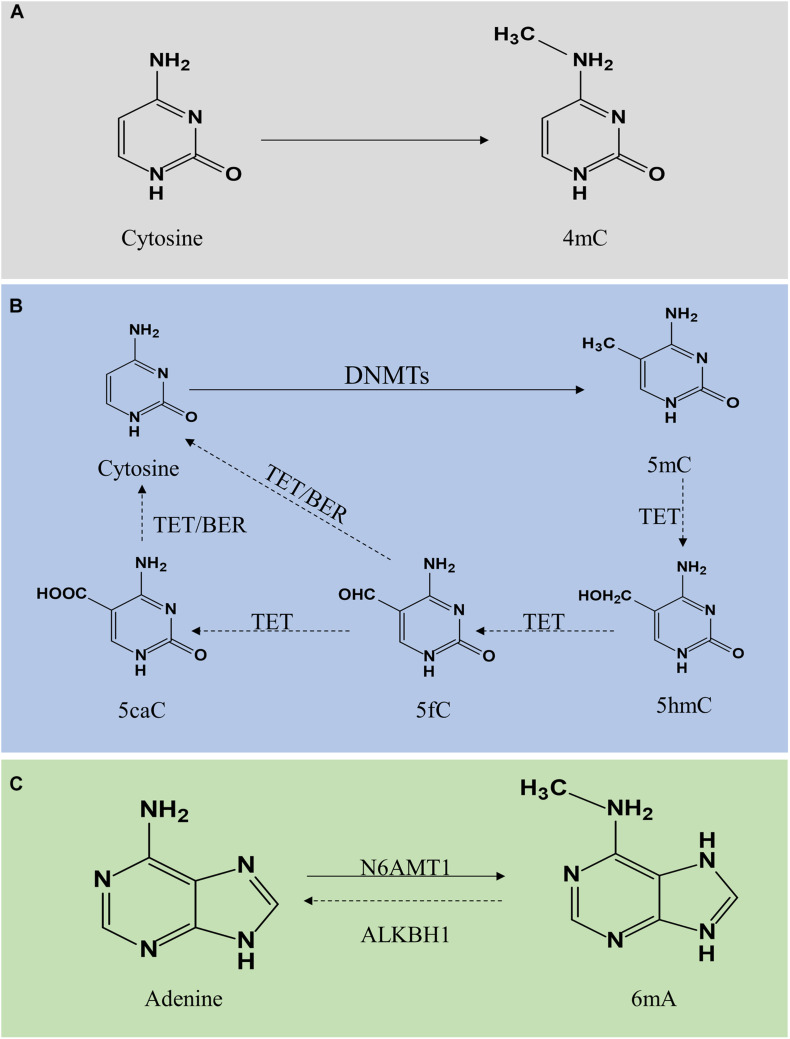
Molecular mechanism of DNA methylation and demethylation. DNA methylation is divided into 4mC, 5mC, and 6mA. Panel **(A)** shows 4mC, a type of DNA methylation in prokaryotes. Panel **(B)** shows 5mC. A cytosine base can be methylated by DNMT1, DNMT3a, and DNMT3b to form 5mC, while TETs catalyze the oxidation of 5mC to 5hmC, 5fC, and 5caC. 5fC and 5caC are modulated by TDG/BER pathway. Panel **(C)** shows 6mA. Adenine is catalyzed by N6AMT1 to form 6 mA. In contrast, ALKBH1 mediates *N*^6^-demethyladenine. 4mC, *N*^4^-methylcytosine; 5mC, *N*^5^-methylcytosine; 6mA, *N*^6^-methyladenine; DNMTs, DNA methyltransferases; TETs, 10–11 translocations; 5hmC, 5-hydroxymethylcytosine; 5fC, 5-formylcytosine; 5caC, 5-carboxylcytosine; BER, base excision repair.

DNA methylation is a modification of DNA. In 1942, Waddington first proposed the concept of “epigenotype,” which was used to explain the complex progression process between genotype and phenotype (Waddington, 2012). To date, it is generally accepted that epigenetics primarily focuses on regulating gene expression, including DNA methylation, histone modification, non-coding RNA modification, chromatin remodeling, gene imprinting, etc. Here, we majorly focus on DNA methylation and its vital role in vascular aging-related diseases, including cardiovascular disease (CVD), cerebrovascular disease, and kidney diseases. DNA methylation regulates gene expression by recruiting proteins involved in gene repression or inhibiting the binding of transcription factors to DNA (Moore et al., 2013). DNA methylation is a dynamically reversible process modulated by methyltransferases and demethyltransferases (Chen and Riggs, 2011). There exist three forms of DNA methylation, including *N*^4^-methylcytosine (4mC), *N*^5^-methylcytosine (5mC), and *N*^6^-methyladenine (6mA) (Ratel et al., 2006) (Figure 1).

### *N*^5^-Methylcytosine (5mC)

*N*^4^-methylcytosine (4mC) modification principally exists in the DNA of bacteria, hence, a subject ignored by this review (Ehrlich et al., 1985, 1987). DNA methylation mainly occurs on the CpG dinucleotide in vertebrates. In mammals, global DNA methylation is a dynamic process, thus there exist DNA methylation and demethylation. 5mC is generated by DNA methyltransferases (DNMTs) through transfer a methyl group from *S*-adenyl methionine to the fifth carbon atom of the cytosine (Moore et al., 2013; Lyko, 2018). The DNMTs family includes DNMT1, DNMT2, DNMT3a, DNMT3b, and DNMT3L (Lyko, 2018). The patterns of 5mC are categorized into two major groups, including maintenance methylation and *de novo* methylation. DNMT1 is implicated in the DNA methylation through copy 5mC from parental DNA strand onto the newly synthesized daughter strand during DNA replication, thus, DNMT1 is known as maintenance DNMT (Mortusewicz et al., 2005; Nelissen et al., 2011). The *de novo* DNMTs included DNMT3a and DNMT3b can generate methylation in unmethylated DNA (Law and Jacobsen, 2010; Moore et al., 2013). Generally, DNA methylation inhibits gene expression through two different mechanisms. One is on the basis of intervene the binding of transcription factors included E2F or CREB by DNA methylation to represses gene transcription. On the other hand, DNMTs interact with methyl-CpG binding domain proteins to suppress transcription through establish a repressive chromatin environment (Bogdanović and Veenstra, 2009). DNA hypermethylation is strongly associated with the development of vascular aging-related disorders. Besides, DNA demethylation refers to two different pathways, including active demethylation and passive demethylation. Active DNA demethylation involves the ten-eleven translocations (TETs) induced methylated base oxidation and the activation induced deaminase induced methylated or a nearby base deamination, respectively (Bochtler et al., 2017). Activation induced deaminase deaminates cytosine to uracil. Additionally, several sources of evidence confirmed that 5mC can be converted into 5-hydroxymethylcytosine (5hmC), 5-formylcytosine (5fC), and 5-carboxylcytosine (5caC) under the activation of TETs (Rasmussen and Helin, 2016; Wu and Zhang, 2017). The level of TETs is tightly associated with DNA methylation. A high generation of TETs significantly alleviated the level of 5mC while a lack of TETs induced DNA hypermethylation. TET-thymine DNA glycosylase (TDG)-base excision repair (BER) mechanism is involved in regulating active DNA demethylation (Weber et al., 2016). Additionally, passive DNA demethylation occurs in DNA replication via the dilution of methylation marks (Ooi and Bestor, 2008). DNMT1 is implicated in the process of maintenance methylation, inhibiting the expression or activation of DNMT1 reduces the level of DNA methylation.

### *N*^6^-Methyladenine (6mA)

DNA *N*^6^-methyladenine (6mA) was previously considered the most prevalent form of DNA methylation in prokaryotes (Xiao et al., 2018). Surprisingly, recent reports suggested that 6mA also exists in eukaryotes, including *Caenorhabditis elegans* (Greer et al., 2015), *Drosophila* (Zhang G. et al., 2015), and *Chlamydomonas* (Fu et al., 2015). In *C. elegans*, 6mA is mediated by DAMT-1 and NMAD-1 (Greer et al., 2015). In *Drosophila*, DMAD is implicated in the regulation of 6mA demethylation (Zhang G. et al., 2015). In addition, a study revealed that 6mA was extensively present in the human genome and enriched in the coding region, modulating gene transcription activation (Xiao et al., 2018). Furthermore, human genome 6mA modification is modulated by the methyltransferase N6AMT1 while *N*^6^-demethyladenine is mediated by ALKBH1. Several studies indicated that 6mA was involved in human diseases with controversy, thus, further exploration and in-depth studies on the roles of 6mA in the field of mammalian biomedicine are necessary to address the controversy.

## The Role and Mechanism of DNA Methylation in Vascular Aging

**FIGURE 2:**
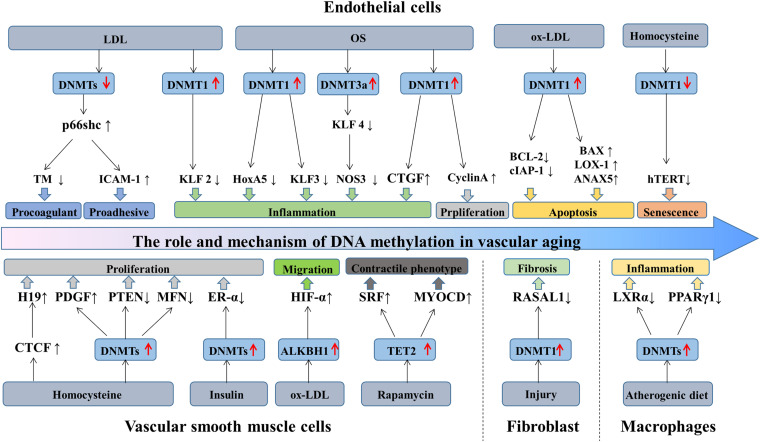
The role and mechanism of DNA methylation in vascular aging. During aging, the structure and function of vascular cells change, causing ECs dysfunction, VSMCs proliferation and migration, fibroblasts differentiation, and macrophages inflammation. LDL stimulates p66shc expression by downregulating the activity of DNMTs and mediating the expression of ICAM-1 and TM, eventually exacerbating ECs dysfunction. Besides, LDL inhibits KLF2 expression via increasing DNMT1 activity, causing ECs inflammation. OS-treated ECs promotes DNMT1 activity and inhibits the generation of HoxA5 and KLF3. OS regulates KLF4, CTGF, and cyclinA methylation patterns by promoting DNMTs activity, causing ECs inflammation and proliferation. Ox-LDL triggers ECs apoptosis through the hypermethylation of cIAP-1 and BCL2 and the hypomethylation of BAX, LOX-1, and ANXA5. Homocysteine promotes ECs senescence by reducing hTERT expression via DNA methylation. Additionally, homocysteine increases CTGF and PDGF expression and decreases PTEN and MFN expression, mediating VSMCs proliferation. Insulin promotes VSMCs proliferation via ER-α hypermethylation. Ox-LDL promotes VSMCs migration by increasing HIF-1α generation through ALKBH1. TET2 overexpression promotes VSMCs differentiation by the upregulation of SRF and MYOCD expression. Besides, in response to injury, fibroblast activation through DNMT1 upregulation and *RASAL1* hypermethylation. Furthermore, the hypermethylation of *LXR*α and *PPAR*γ*1* induces macrophages inflammation. DNMTs, DNA methyltransferases; TET, 10—11 translocations; LDL, low-density lipoprotein; OS, oscillatory shear stress; ECs, endothelial cells; VSMCs, vascular smooth muscle cells.

Aging is a decline of the biological system, accompanied by a decrease in function. Cell senescence is a permanent state of cell cycle arrest (Khor and Wong, 2020), causing tissue dysfunction and closely associated with aging-related diseases (Bitar, 2019). Accumulating evidence suggested that DNA methylation plays a significant role in regulating ECs, VSMCs, fibroblasts, and macrophages functions, and is implicated in the process of vascular aging and related disorders. Recent studies have identified a series of genes regulated through DNA methylation in the initiation and development of vascular aging (Table 1). This section primarily focuses on the mechanisms and roles of DNA methylation in the functions of ECs and VSMCs (Figure 2).

**TABLE 1:** DNA methylation in vascular aging.

Vascular cells	Genes	Methylation status	Functions	References
ECs	*KLF2*	Hyper	Pro-inflammation	Kumar et al., 2013
	*KLF4*	Hyper	Pro-inflammation	Jiang et al., 2014
	*SMAD7*	Hyper	Pro-inflammation	Wei et al., 2018
	*HoxA5*	Hyper	Pro-inflammation	Dunn et al., 2014
	*p66shc*	Hypo	Pro-coagulant	Kim et al., 2012
	*CTGF*	Hypo	Pro-inflammation	Zhang et al., 2017
	*ANXA5, BAX, CASP3, LOX-1*	Hypo	Pro-apoptosis	Mitra et al., 2011
	*BCL2, cIAP-1*	Hyper	Anti- apoptosis	Mitra et al., 2011
	*cyclin A*	Hyper	Pro-proliferation	Zhang et al., 2017
	*hTERT*	Hypo	Pro-senescence	Zhang D. et al., 2015
VSMCs	*MFN2*	Hyper	Pro-proliferation	Xu L. et al., 2019
	*PTEN*	Hyper	Pro-proliferation	Ma et al., 2018
	*ER-*α	Hyper	Pro-proliferation	Min et al., 2016
	*PDGF*	Hypo	Pro-proliferation	Zhang et al., 2012
	*HIF-1*α	Hypo	Pro-migration	Wu et al., 2019
	*MYOCD, SRF, MYH11*	Hyper	Pro-differentiation	Liu et al., 2013
Fibroblasts	*RASSF1A*	Hyper	Fibrosis	Tao et al., 2014a
	*RASAL1*	Hyper	Fibrosis	Bechtel et al., 2010
	*BMP-7*	Hyper	Fibrosis	Xu et al., 2015a
	*COL1A1*	Hypo	Fibrosis	Pan et al., 2013
Macrophages	*PPAR*γ*1*	Hyper	Pro-inflammation	Yang et al., 2014
	*PSTPIP2*	Hyper	Pro-inflammation	Luz et al., 2018
	*LXR*α	Hyper	Pro-inflammation	Cao et al., 2014

*EC, endothelial cells; VSMC, vascular smooth muscle cells.*

### DNA Methylation and ECs Functions

Vascular endothelium is mainly composed of ECs, a barrier between blood and tissues. Endothelial dysfunction is caused by a variety of stimuli such as oxidized low-density lipoprotein (ox-LDL), hypoxia, shear stress, or inflammatory factors (Ni et al., 2020). DNA methylation modulates gene expression and mediates ECs biology in the development of its aging.

#### ECs Functions

Endothelial cells play a crucial role in maintaining vascular homeostasis (Gori, 2018). ECs regulate vasoconstriction and relaxation (Krüger-Genge et al., 2019), involved in physiological processes, including blood coagulation, angiogenesis, and metabolism. Besides, ECs cooperate with VSMCs in modulating blood flow to tissues (Michiels, 2003). ECs aging are characterized by endothelial dysfunction (Jia et al., 2019). Aging causes changes in vascular EC-mediated vasoconstriction and dilation, integrity destruction, increased permeability, destroying the blood–brain barrier, and impaired angiogenesis (Papapetropoulos et al., 1997). Furthermore, ECs senescence stimulates mitochondrial dysfunction and reactive oxygen species (ROS) accumulation, which further aggravates vascular senescence (Ungvari et al., 2008).

#### DNA Methylation and ECs

Generally, DNA methylation is altered in ECs during aging and upon exposure to stimuli such as shear stress, hypoxia, or ox-LDL. DNA hypermethylation and hypomethylation can result in inhibition and stimulation of gene transcription in ECs in response to injury (Levy et al., 2017). Quite a number of evidence highlighted that DNA methylation is involved in mediating the biological processes of ECs, including inflammation, proliferation, senescence, and apoptosis.

Endothelial cells inflammation is tightly linked with vascular disorders. DNMTs promote ECs inflammation by regulating the methylation level of pertinent gene promoter regions. Under the stimulation of low-density lipoprotein (LDL), *KLF2* promoter region was hypermethylated, arousing a down-expression of KLF2 in ECs and causing ECs inflammation and thrombogenesis (Kumar et al., 2013). In addition, the methylation level of relevant genes includes *KLF4* (Jiang et al., 2014), *SMAD7* (Wei et al., 2018), *HoxA5* (Dunn et al., 2014), and *CTGF* (Zhang et al., 2017) are participating in ECs inflammation and aging-related vascular diseases.

Endothelial cells proliferation and differentiation are modulated by DNA methylation. *In vivo*, DNMTs promote the hypermethylation of cell cycle regulator cyclin A, stimulating the proliferation and inflammation of ECs. Besides, endothelial nitric oxide synthase (eNOS) expression in ECs senescence declines, leading to a down-expression of NO and impairing vasodilation (Cau et al., 2012). The generation of eNOS can be regulated by DNA methylation (Chan et al., 2004). Moreover, inhibiting DNA methylation in the *eNOS* promoter region induces the differentiation of human embryonic liver cells into ECs (Lagarkova et al., 2008). In addition, ROS accumulated in aging ECs causes alternation in DNA methylation by changing DNMTs activity and DNA damage (O’Hagan et al., 2011; Tabaei and Tabaee, 2019).

Endothelial cells senescence and apoptosis arouse endothelial dysfunction. Under the condition of ox-LDL, the proapoptosis-related genes such as *LOX-1, ANXA5*, *BAX*, and *CASP3* are activated due to the hypomethylation of its promoter region. In contrast, the anti-apoptotic *BCL2* and *cIAP-1* genes are down-regulated by the DNA hypermethylation, eventually resulting in ECs apoptosis (Mitra et al., 2011). Besides, homocysteine promotes ECs senescence through DNA hypomethylation of *hTERT* (Zhang D. et al., 2015).

### DNA Methylation and VSMCs Functions

Vascular smooth muscle cells, the primary cells of vascular media, mediating blood flow and pressure via vascular contraction and relaxation. Environmental stresses such as oxidative stress, aging, or inflammation stimulate VSMCs contractile state switching into a proliferative and migratory synthetic phenotype, causing cell inflammation, proliferation, and migration. To date, multiple publications supported that DNA methylation plays a role in regulating VSMCs gene expression and VSMCs proliferation, migration, senescence, and apoptosis in response to vascular disorders.

#### VSMCs Functions

Vascular smooth muscle cells are implicated in maintaining the structural integrity and physiological function of blood vessels, regulating blood pressure and controlling vascular contraction and relaxation (Frismantiene et al., 2018; Chi et al., 2019). Besides, VSMCs and extracellular matrix are the primary regulators of arterial contraction tension and vascular tension (Wang G. et al., 2015; Chi et al., 2019). With aging, VSMCs suffer from mechanical stimulation, chronic inflammation, calcification, epigenetic events, etc. (Lacolley et al., 2018). VSMCs switch into synthetic phenotype, leading to hypertrophy of the vascular wall and collagen deposition, which is pertinent to vascular aging-related diseases.

#### DNA Methylation and VSMCs

DNA methylation is implicated in the modulation of VSMCs proliferation, migration, differentiation, and calcification. The proliferation of VSMCs is one of the primary features of vascular aging-related diseases, and DNA methylation is associated with the regulation of gene transcription controlling cell proliferation. A large amount of evidence revealed that DNMTs inhibited gene expression by promoting DNA methylation and regulating the proliferation of VSMCs. DNA hypermethylation of *MFN2* (Xu L. et al., 2019), *PTEN* (Ma et al., 2018), and *ER-*α (Min et al., 2016) causes the corresponding low expression of MFN2, PTEN, and ER-α and promotes the proliferation of VSMCs. On the contrary, DNA demethylation of *PDGF* increases PDGF mRNA and protein expression and promotes the proliferation and migration of VSMCs (Zhang et al., 2012). Additionally, the hypomethylation of *HIF-1*α causes VSMCs proliferation and migration as well (Wu et al., 2019).

Vascular smooth muscle cells have apparent plasticity. TET2, a key enzyme in the DNA demethylation pathway, participates in regulating the differentiation of SMCs. A study demonstrated that in the case of vascular injury and vascular disorders, the expression of TET2 was downregulated, whereas the *MYOCD*, *SRF*, and *MYH11* were hypermethylated, resulting in SMCs differentiation (Liu et al., 2013).

The phenotypic modulation of SMCs triggers vascular disorders, including vascular calcification and atherosclerosis. Accumulating evidence reported that the contraction and synthetic phenotypes of VSMCs were mediated by DNA methylation. Furthermore, extracellular matrix mediates the phenotypic transition of SMCs via DNA methylation (Liu R. et al., 2015). Conversely, the DNMTs inhibitor 5-aza-2′-deoxycytidine (5Aza) reduces the DNA methylation level of the *ALP* in VSMCs and promotes the expression and activity of alkaline phosphatase, inducing vascular calcification (Azechi et al., 2014).

### DNA Methylation and Vascular Fibroblasts Functions

The vascular adventitia is primarily composed of fibroblasts. Fibroblasts are thought to exhibit a broad range of physiological functions. Fibroblasts increase vasa vasorum-associated neointima formation and macrophage recruitment by enhancing the expression of vascular endothelial growth factors (Li X. D. et al., 2020). Besides, fibroblasts are involved in vascular remodeling, inflammation, and neointimal formation in vascular disorders (An et al., 2015). Moreover, fibroblasts play a role in providing a supporting framework of the vessel wall through generating and secreting fibrillar collagens, the main components of the adventitial extracellular matrix (Stenmark et al., 2013). Additionally, in response to injury, fibroblasts generate accumulative extracellular matrix, ultimately lead to organ dysfunction, such as liver cirrhosis and chronic renal failure (Thannickal et al., 2014).

It has been identified that aging is a risk factor for fibrosis. DNA methylation is involved in modulating fibroblasts activity. RASSF1A, a regulatory tumor inhibitor, is downregulated by DNMT3a in cardiac fibrosis and fibroblasts activation. Mechanistically, *RASSF1A* hypermethylation promoted cardiac fibrosis through the activation of the Ras/ERK signal pathway (Tao et al., 2014a). Besides, the hypermethylation of *RASAL1* induced by DNMT1 is consistently linked with fibroblast activation and kidney fibrosis (Bechtel et al., 2010). Aberrant hypermethylation of several genes includes *RASSF1A, RASAL1, BMP7*, and *COL1A1* are tightly correlated with fibroblast activation.

### DNA Methylation and Vascular Macrophages Functions

Monocytes/macrophages, essential components of the immune system, are heterogeneous and exhibit a vital role in modulating inflammatory responses and vascular functions (Shapouri-Moghaddam et al., 2018). Accumulating studies revealed that monocytes and macrophages exert indispensable roles in the onset and development of chronic inflammatory disorders such as atherosclerosis, diabetes, and cancer. Commonly, macrophages exist in two distinct subpopulations includes classically activated or M1 macrophages and alternatively activated or M2 macrophages. M1 macrophages secrete numerous pro-inflammatory cytokines, such as interleukin-1α (IL-1α), IL-1β, IL-6, IL-12, and cyclooxygenase-2 whereas M2 macrophages generate anti-inflammatory cytokines such as IL-10 and transforming growth factor-β (Shapouri-Moghaddam et al., 2018). Macrophages interact with major histocompatibility complex molecules to present antigen. Moreover, macrophages are implicated in possessing the phagocytosis of pathogens, debris, and dead cells.

Increasing evidence indicated that DNA methylation functions as a significant regulator of monocyte-macrophage phenotypes and functions (Shapouri-Moghaddam et al., 2018). A study compared genome-wide DNA methylation among monocytes and macrophages and found that differential DNA methylation was presented in monocyte to macrophage differentiation, majorly restricted to very short regions (Dekkers et al., 2019). In addition, the loss of DNA methylation was pronounced during monocytes to dendritic cells differentiation and modulated by TET2 (Klug et al., 2013). Furthermore, macrophages polarization and inflammation are regulated by DNMT3b. The expression of DNMT3b was significantly lower in M2 macrophages compared with M1 macrophages. Notably, DNMT3b knockdown causes macrophage polarization to M2 phenotype and inhibits inflammation, whereas DNMT3b overexpression promotes to M1 phenotype and aggravates inflammation. Mechanistically, DNMT3b is implicated in silencing the promoter of *PPAR-*γ*1* (Yang et al., 2014). Besides, TET2 suppresses the expression of IL-6 in mouse macrophages (Hoeksema and de Winther, 2016).

## DNA Methylation in Vascular Aging-Related CVD

Cardiovascular disease is the leading cause of death among the elderly. By 2050, the global population aging above 60 years will approximate 2.1 billion (Wyss-Coray, 2016). Therefore, the incidence and mortality of CVD will have a gradual increase. Vascular aging is a major trigger of many vascular disorders, while vascular cell senescence shows significant effect on the progression of CVD. Increasing evidence revealed the role of epigenetic mechanisms in vascular aging-related diseases. It’s well-known that abnormal DNA methylation and DNMTs expression are tightly associated with vascular diseases (Hiltunen et al., 2002). In this part, we mainly focus on the roles and mechanisms of DNA methylation in aging-related atherosclerosis, hypertension, and other vascular disorders. Besides, we discuss the connection between clonal hematopoiesis and CVD.

### Atherosclerosis

Atherosclerosis is a vascular disorder with complicated processes comprising ECs dysfunction, VSMCs proliferation and migration, macrophages inflammation, and collagen matrix accumulation. Age is a fundamental risk factor for atherosclerosis (Uryga and Bennett, 2016). DNA methylation plays a vital role in the initiation and progression of atherosclerosis. Besides, DNMTs act as regulators of vascular structure and functions (Table 2).

**TABLE 2:** DNA methylation in vascular aging-related atherosclerosis.

Genes	Methylation Status	Sample source	References
*KLF2*	Hyper	ECs	Kumar et al., 2013
*KLF4*	Hyper	ECs	Jiang et al., 2014
*HoxA5, KLF3*	Hyper	ECs	Dunn et al., 2014, 2015
*p66shc*	Hypo	ECs	Kim et al., 2012
*BAX, LOX-1, CASP3*	Hypo	ECs	Mitra et al., 2011
*cIAP-1, BCL2*	Hyper	HUVEC	Mitra et al., 2011
*HIF1*α	Hypo	VSMCs	Wu et al., 2019
*MYOCD, SRF, MYH11*	Hyper	VSMCs	Liu et al., 2013
*p53, PTEN, MFN2*	Hyper	VSMCs	Ma et al., 2017
*PDGF*	Hypo	VSMCs	Ma et al., 2017
*CTCF*	Hypo	HUVSMCs	Li et al., 2009
*PPAR*γ*1*	Hyper	Macrophages	Yang et al., 2014
*PSTPIP2*	Hyper	Macrophages	Luz et al., 2018
*LXR*α	Hyper	Macrophages	Cao et al., 2014
*ABCA1, TIMP1, ACAT1*	Hyper	Peripheral blood	Ma et al., 2016
*SMAD7*	Hyper	Peripheral blood	Wei et al., 2018
*DDAH2*	Hyper	Peripheral blood	Niu et al., 2014
*IL-6*	Hypo	Peripheral blood	Yang Q. et al., 2016
*LDLR*	Hypo	Peripheral blood	Guay et al., 2013
*eNOS*	Hyper	Artery	Chan et al., 2004
*ER*β	Hyper	Artery	Kim et al., 2007
*15-LO*	Hypo	Artery	Liu et al., 2004
*ER*α	Hyper	Atherosclerotic plaque	Post et al., 1999
*MAP4K4, ZEB1, FYN*	Hyper	Atherosclerotic plaque	Yamada et al., 2014
*Foxp3*	Hyper	Atherosclerotic plaque	Zhu et al., 2019
*HECA, EBF1, NOD2*	Hypo	Atherosclerotic plaque	Yamada et al., 2014
*MMP9*	Hyper	Macrophages	Fisslthaler et al., 2019
*APOE*	Hyper	Human	Zhang et al., 2019

*EC, endothelial cells; VSMC, vascular smooth muscle cells; HUVSMCs, human umbilical vein smooth muscle cells.*

Endothelial cells senescence are accompanied by ECs dysfunction, decreased angiogenesis, and damaged eNOS activity, responsible for atherosclerosis progression (Colpani and Spinetti, 2019). Additionally, VSMCs are an important part of the fibrous cap and stromal cells of the artery (Chi et al., 2019). Accumulating evidence demonstrated that collagen secretion decreased in the aging of VSMCs, forming unstable fibrous caps (Gardner et al., 2015). VSMCs senescence enhances plaque vulnerability by secreting matrix metalloproteinases degrading the matrix and releases numerous pro-inflammatory cytokines promoting plaque inflammation and impairing the stability of plaques (Wang J. et al., 2015; Grootaert et al., 2018). Besides, Macrophages are defined as key modulators in atherosclerosis with aging and chronic inflammation (Moore and Tabas, 2011). Activated macrophages and foam cells trigger a cascade of inflammatory responses and induce atherosclerotic plaque formation. Notably, macrophages proliferation is the predominant mechanism in atherosclerotic plaques (Xu H. et al., 2019).

Numerous lines of evidence supported that atherosclerosis is manifested by global DNA hypomethylation and regional DNA hypermethylation. The level of DNA methylation in atherosclerotic arteries in rabbit models is lower compared to normal arteries (Laukkanen et al., 1999). Scholars confirmed that aberrant DNA methylation in atherosclerosis influence the transcription of key regulatory genes, inducing the pro-atherosclerotic cell phenotype (Castillo-Díaz et al., 2010). Gene hypermethylation including *ER-*α (Post et al., 1999; Huang et al., 2007), *DDAH2* (Niu et al., 2014), and *Foxp3* (Zhu et al., 2019) are tightly associated with the occurrence and development of atherosclerosis. Additionally, ROS accumulates in the aging of VSMCs and ECs (Chi et al., 2019; Tabaei and Tabaee, 2019), inducing DNA methylation changes via the altered activity of DNMTs and DNA damage, which modulates the formation and development of atherosclerotic plaques.

### Hypertension

Hypertension, defined as average systolic blood pressure ≥140 mm Hg and/or average diastolic blood pressure ≥90 mm Hg, is the leading cause of CVD worldwide (Mills et al., 2020). Aging triggers a functional decline of various organ systems in the body. Vascular aging is significantly linked to hypertension prevalence and mortality among the elderly (Cheng et al., 2017; Donato et al., 2018). Besides, hypertension is an important risk factor for other vascular aging-related diseases, such as dementia, and cognitive decline (Cortes-Canteli and Iadecola, 2020; Fuchs and Whelton, 2020). Emerging documents demonstrated that DNA methylation exhibits a significant impact on hypertension development through modulating gene expression and vascular cell functions (Table 3).

**TABLE 3:** DNA methylation in vascular aging-related hypertension.

Genes	Methylation Status	Functions	References
*sACE*, *ACE-1*	Hyper	RAAS	Rivière et al., 2011
*ACE-2*	Hyper	RAAS	Fan et al., 2017
*Atgr1*α	Hypo	RAAS	Pei et al., 2015
*Atgr1*β	Hypo	RAAS	Bogdarina et al., 2007
*AGT*	Hypo	RAAS	Wang et al., 2014
*AGTR1*	Hypo	RAAS	Fan et al., 2015
*SULF1*, *PRCP*	Hyper	Inflammation	Wang et al., 2013
*IL-6*	Hypo	Inflammation	Mao S. Q. et al., 2017
*IFN-*γ	Hypo	Inflammation	Bao et al., 2018
*TLR2*	Hypo	Chronic inflammation	Mao S. et al., 2017
*EHMT2*	Hypo	Chronic inflammation	Boström et al., 2016
*ADD1*	Hypo	Ionic balance	Zhang et al., 2013
*NKCC1*	Hypo	*Ionic balance*	Cho et al., 2011
*SCNN1B*	Hyper/hypo	Ionic balance	Zhong et al., 2016
*MTHFD1*	Hyper	Hyperhomocysteinemia	Xu M. et al., 2019
*CBS*	Hyper	Hyperhomocysteinemia	Wang et al., 2019
*SHMT1*	Hyper	Hyperhomocysteinemia	Xu G. et al., 2019
*ER*α	Hyper	Vasodilation	Dasgupta et al., 2012
*11*β*HSD2(HSD11B2)*	Hyper	Aldosterone	Alikhani-Koopaei et al., 2004

*RAAS, renin–angiotensin–aldosterone system.*

It has been recognized that endothelial dysfunction contributed to the initiation and progression of hypertension. ECs release endothelin-1, prostacyclin, NO, and other vasoactive substances regulating vasoconstriction and relaxation (Sandoo et al., 2010). The dysfunction of aging ECs creates an imbalance between vasoconstriction and relaxation, leading to an increase in blood pressure (Jia et al., 2019). In addition, VSMCs mediate arterial compliance and total peripheral resistance (Chi et al., 2019). VSMCs senescence stimulates hypertension by the accumulation of oxidative stress and inflammation. Besides, vascular macrophages trigger endothelial dysfunction by increasing the expression of ROS and inflammatory cytokines, leading to vascular oxidative stress and blood pressure elevation (Justin Rucker and Crowley, 2017). The senescence of vascular cells promotes arterial stiffness, causing hypertension (Qiu et al., 2010; Chi et al., 2019).

DNA methylation is involved in hypertension (Gao et al., 2018; Guo et al., 2020; Amenyah et al., 2021). Kazmi et al. investigated the relationship between hypertension and DNA methylation in European men and discovered that 7 CpG sites were related to diastolic blood pressure (Kazmi et al., 2020). In addition, a study surveyed the association between blood pressure and DNA methylation among Europeans, Hispanics, and African Americans and identified 14 relevant CpG sites (Richard et al., 2017). Moreover, DNA methylation of the natriuretic peptide-A gene was decreased in patients diagnosed with hypertension among the Chinese community (Li J. et al., 2020). As we all know, the renin–angiotensin–aldosterone system (RAAS) is vital to the occurrence and progression of hypertension (Drummond et al., 2019). For instance, *AT1aR* promoter region hypomethylation in hypertensive rats upregulates the expression of AT1aR, critical to the development of hypertension (Pei et al., 2015). Administration of angiotensin receptor antagonists hinders the progression of hypertension in the early stages (Bogdarina et al., 2007). Besides, the level of 6mA is decreased in hypertensive mice and rat models, causing phenotypic transformation and migration of VSMCs (Guo et al., 2020).

### DNA Methylation and Other Vascular Aging-Related CVD

Heart failure (HF) is a clinical syndrome caused by impairment of the systolic and diastolic functions. With the aging population, the incidence and mortality of HF are gradually increasing. In developed countries, the prevalence of HF among the elderly aged above 65 is approximately 11.8% (Groenewegen et al., 2020). Aging induces cardiovascular senescence and myocardial fibrosis (Horn and Trafford, 2016), leading to cardiac dysfunction and promoting HF progression (Triposkiadis et al., 2020). Notably, cardiovascular senescence, atherosclerosis (Triposkiadis et al., 2019), hypertension (Fuchs and Whelton, 2020), and ischemic cardiomyopathy (Napoli et al., 2020) are critical risk factors for HF (Li H. et al., 2020). DNA hypermethylation regulates cardiometabolism by destroying nuclear respiratory factor 1 dependent oxidative metabolism (Pepin et al., 2019). Additionally, in HF patients, the expression of DNMT3a and DNMT3b were upregulated, inhibiting the mRNA levels of several oxidative metabolism genes (Pepin et al., 2019). DNMT3b knockout can induce cardiac contractile insufficiency, ventricular wall thinning, and accelerating the deterioration of contractile function during HF (Vujic et al., 2015). Moreover, aberrant DNA methylation in dilated cardiomyopathies patients is associated with significant *ADORA2A* and *LY75* mRNA expression changes, but not in *HOXB13* and *ERBB3* (Haas et al., 2013).

Acute myocardial infarction (AMI) primarily occurs based on atherosclerotic stenosis of the coronary arteries. It is attributed to certain triggers of plaque rupture, including platelets gathering on the surface of the ruptured plaque, forming a thrombus that suddenly blocks the lumen of the coronary artery, leading to myocardial ischemic necrosis. The hypermethylation of the *ABO* gene is seemingly linked with an increased risk of AMI in Pakistani (Yousuf et al., 2020). Besides, the hypomethylation of *ZBTB12* gene and *LINE-1* gene are early biomarkers of MI in peripheral blood white cells (Guarrera et al., 2015). Besides, the methylation of *ZFHX3* and *SMARCA4* are independently and significantly related to MI (Nakatochi et al., 2017).

Coronary heart disease (CHD) is a heart condition caused by atherosclerotic lesions in the coronary arteries, causing ischemia, hypoxia or necrosis of the myocardium. DNA methylation is connected with the risk of future CHD. *ATP2B2, GUCA1B, CASR*, and *HPCAL1* are genes regulating calcium modulation (Agha et al., 2019). DNA methylation and hydroxymethylation among the elderly CHD patients were significantly upgraded. Report supported that a lower methylation level in the *SOAT1* gene might enhance the risk of CHD (Abuzhalihan et al., 2019).

Cardiac fibrosis is defined as the accumulation of extracellular matrix proteins in the cardiac interstitium (Tao et al., 2014b). Several studies indicated that DNA methylation is related to the onset and development of tissue fibrosis. Increased *RASAL1* and *RASSF1A* promoter methylation is involved in cardiac fibrosis (Tao et al., 2014a; Xu et al., 2015b). Besides, transforming growth factor-beta 1 can induce *COL1A1* demethylation and collagen type I expression by suppressing the generation and activity of DNMT1 and DNMT3a (Pan et al., 2013).

### Clonal Hematopoiesis and CVD

Human aging is linked with an increased frequency of somatic mutations in hematopoietic system. This clonal hematopoiesis is associated with CVD (Evans et al., 2020). Age-related clonal hematopoiesis is major occurred in DNMT3a and TET2 and is associated with CVD (Jaiswal et al., 2014). TET2, highly expressed in murine macrophage differentiation, inhibits inflammatory gene expression in macrophages, reducing macrophages inflammation. In contrast, TET2-deficient macrophages affect phenotype of macrophages to promote chronic inflammation in vasculature, resulting in the progression of CVD (Cull et al., 2017). Studies in LDL receptor-deficient mice with TET2-deficient cells induce an increase in atherosclerotic plaque size. Besides, TET2-deficient macrophages enhance the secretion of interleukin-1β (IL-1β), regulated by inflammasome NLRP3 (Fuster et al., 2017). Additionally, DNMT3a is implicated in regulating inflammatory pathways and macrophage functions. Accumulating evidence revealed that hematopoietic DNMT3a mutation can promote HF through exacerbating inflammatory responses. HF Patients with monocytes carrying DNMT3a mutations show an increasing expression of inflammation genes, including IL-1B, IL-6, IL-8, NLRP3, CCL3, and CCL4, which may be contributing to exacerbating HF (Abplanalp et al., 2021). In addition, mice with mutations in TET2 or DNMT3a following an infusion of angiotensin II diminished cardiac function, increased fibrosis and inflammation (Sano et al., 2018). IL-1β was upregulated in TET2-deficient cells, while CXCL1 and CXCL2 were upregulated in DNMT3a-deficient cells. Research between clonal hematopoiesis and CVD is very much in its infancy. To date, only atherosclerosis and HF have been evaluated with clonal hematopoiesis in TET2 or DNMT3a mutation. Further studies should examine the role of clonal hematopoiesis in CVD beyond atherosclerosis and HF (Evans et al., 2020).

## DNA Methylation and Vascular Aging-Related Cerebrovascular Diseases

Cerebrovascular diseases are a group of diseases causing damage to brain tissue due to blood circulation disorder in the brain. Arterial stiffness and vascular aging trigger cerebrovascular dysfunction and blood–brain barrier contraction. Subsequently, a series of cerebrovascular diseases occur (Toth et al., 2017; Thorin-Trescases et al., 2018; Kalaria and Hase, 2019). Besides, small blood vessel diseases are common in the aging process, manifested as brain and parenchymal microcirculation changes (De Silva and Faraci, 2020), decreasing cerebral blood flow and damaging the blood–brain barrier, eventually lead to an aging-related functional decline of the brain (Iadecola et al., 2019). DNA methylation regulates various cerebrovascular diseases, such as stroke, dementia, and AD (Table 4).

**TABLE 4:** DNA methylation in vascular aging-related cerebrovascular diseases.

Diseases	Genes	Methylation status	References
Stroke	*CBS*	Hyper	Wang et al., 2019
	*TM*	Hyper	Yang Z. et al., 2016
	*ApoE*	Hyper	Zhang et al., 2019
	*ABCG1*	Hyper	Qin et al., 2019
	*CDKN2B*	Hyper	Zhou et al., 2017
	*PPM1A*	Aberrant	Gallego-Fabrega et al., 2016a
	*TRAF3*	Hypo	Gallego-Fabrega et al., 2016b
	*LINE-1*	Hypo	Baccarelli et al., 2010
	*MTRNR2L8*	Hypo	Shen et al., 2019
AD	*ApoE*	Hyper	Rajan et al., 2015
	*ANK1*	Hyper	Lunnon et al., 2014
	*RHBDF2*	Hyper	De Jager et al., 2014
	*ABCA7*	Hyper	Yamazaki et al., 2017
	*RPL13,CDH23*	Hyper	Prasad and Jho, 2019
	*ANKRD30B*	Hyper	Hua et al., 2019

*AD, Alzheimer’s disease.*

### Stroke

Stroke is a leading cause of death and disability globally. Endothelial dysfunction and macrophages polarization contribute to stroke (Blum et al., 2012). Besides, the vascular stiffness of the elderly population increases with aging. Studies indicated that carotid artery stiffness is a crucial factor in the onset and development of stroke (Mattace-Raso et al., 2006; van Sloten et al., 2015). Pulse wave velocity is used to analyze age-related changes in vascular structure and function as well as to evaluate the endothelial function and vascular stiffness (Stoner et al., 2012). In a cohort study based on a Chinese community population, assessing vascular aging might help stroke risk assessment (Yang et al., 2019). DNA methylation changes with age and is linked to stroke during aging (Soriano-Tárraga et al., 2014). In contrast with healthy subjects, the methylation level of the *TP53* promoter region increased among stroke patients (Wei et al., 2019). Besides, the methylation of *MTRNR2L8* is a potential therapeutic target for stroke (Shen et al., 2019).

### Dementia and AD

Dementia is a decline in intelligence that severely disrupts daily life (Iadecola et al., 2019). The aging population is crucial to age-related cognitive abilities and important public health challenges (Deak et al., 2016). Research estimated that by 2050, 115 million people will be globally diagnosed with dementia (Prince et al., 2013). Endothelial dysfunction and damage might cause neurovascular dysfunction, resulting in microvascular thrombosis and destruction of the blood–brain barrier (Yamazaki et al., 2016). AD is the most prevalent cause of dementia, while age is an independent risk factor for AD (Delaye et al., 1975), and it has been proved that the progression of AD is tightly related to the alteration of DNA methylation (Qazi et al., 2018; Huo et al., 2019). Although AD is a neurodegenerative disease, it is also attributed to cerebrovascular aging (Iturria-Medina et al., 2016), and most AD patients suffer from Aβ amyloid angiopathy (Love and Miners, 2016). Studies suggest that genes including *ANK1* (Lunnon et al., 2014), *RHBDF2* (De Jager et al., 2014), *ABCA7* (Yamazaki et al., 2017), *RPL13*, and *CDH23* were hypermethylated in AD (Prasad and Jho, 2019). Among them, *ANK1*, *ABCA7*, and *RHBDF2* hypermethylation were associated with the formation of Aβ plaques. Higher DNA methylation levels in the promoter region of *APOE* promote the odds of dementia and AD (Karlsson et al., 2018). Besides, *ANKRD30B* is hypermethylated among AD patients, further implying that DNA methylation regulates the progression of AD (Semick et al., 2019).

## DNA Methylation and Vascular Aging-Related Kidney Diseases

Chronic kidney disease (CKD) refers to abnormalities in chronic kidney structure or function caused by various reasons for more than 3 months (Ingrosso and Perna, 2020). CKD is characterized by the development of renal fibrosis subsequent renal failure. Macrophage polarization and fibroblasts differentiate into myofibroblasts are central processes of renal fibrosis (Tang et al., 2019). CKD and renal fibrosis affect approximately 10% of the world’s population and half of the adults aged over 70 (Humphreys, 2018). DNA methylation changes in the renal cortex of patients with CKD (Chu et al., 2017). Besides, a study found that a low DNA methylation in patients with CKD (Zinellu et al., 2017). DNA methylation of genes is linked with CKD and renal fibrosis, including *PTPN6, CEBPB, EBF1, Klotho* (Chu et al., 2017; Yin et al., 2017), *SMAD7* (Yang et al., 2020), *sFRP5* (Yu et al., 2017), and *RASAL1*. Notably, DNA methylation inhibits erythropoietin expression, causing anemia, a prevalent complication of CKD (Yin and Blanchard, 2000).

## DNA Methylation as a Diagnostic Tool and Therapeutic Target in Vascular Aging-Related Diseases

Aging is an inevitable process and significantly associated with many vascular aging-related disorders. Vascular aging is the structural and functional changes of the vasculature, including vascular cells senescence, inflammation, oxidative stress, and calcification (Ding et al., 2020). With aging, there is a gradual increase in the prevalence and mortality of vascular aging-related diseases. A high percentage of vascular aging-related diseases progress to functional failure because no available drugs can reverse vascular aging progression. Therefore, there is an urgent need to discover tools and methods for the early diagnosis and treatment of vascular aging-related diseases. Improving vascular cell senescence might ameliorate vascular aging and related diseases, providing novel ideas for clinical research as well as new prevention and treatment for vascular aging-related diseases.

### DNA Methylation as a Diagnostic Tool in Vascular Aging-Related Diseases

The evidence mentioned above reveals that aberrant DNA methylation modification exists in aging-related vascular diseases and might be a potential biomarker for the diagnosis and prognosis of vascular aging-related diseases (Liu and Tang, 2019). Gene at DNA methylation status based on monocyte/macrophage might work as a diagnostic biomarker for clinical application (Bakshi et al., 2019). Notably, hyperhomocysteine is associated with CVD, potentially influencing DNA methylation modification, suggesting that DNA methylation is a biomarker for the increased risk of CVD (Kim et al., 2010). For example, *BRCA1* and *CRISP2* specific site methylation changes are associated with atherosclerosis, indicating that differentially methylated regions of *BRCA1* and *CRISP2* emerge as biomarkers for CVD (Istas et al., 2017). Moreover, the DNA methylation levels of *LINE-1* in the blood of 742 elderly men and discovered that *LINE-1* hypomethylation is linked to elevated serum vascular cell adhesion molecule-1, related to atherosclerosis progression and high cardiovascular risk (Baccarelli et al., 2010). Additionally, Additionally, DNMT3a expression can be used as novel diagnostic biomarkers for cerebrovascular aging-related diseases (Martínez-Iglesias et al., 2020). Besides, the methylation status of *LINE-1* and *MTRNR2L8* act as epigenetic biomarkers in stroke patients (Baccarelli et al., 2010; Shen et al., 2019). Specific gene methylation of diseases, combined with changes in DNMTs and TETs levels, are diagnostic and prognostic biomarkers providing broad clinical prospects. Further studies should concentrate on the clinical application of DNA methylation as a potential biomarker of vascular aging-related diseases.

### DNA Methylation-Based Therapies in Vascular Aging-Related Diseases

DNA methylation is a dynamically reversible process, providing potential therapeutic targets for delaying or enhancing vascular aging-related diseases. Several agents targeting epigenetic modulators are currently undergoing preclinical or clinical evaluation to treat cancers and might have potential applications in treating cardiovascular events (Gorabi et al., 2020). Drugs that inhibit DNMTs have identified to be promising treatment strategies for many vascular disorders. There are many natural and synthetic compounds able to suppress DNMT activity (Table 5).

**TABLE 5:** DNA methylation-based drugs for vascular aging-related diseases.

Compounds	Nutrients/drugs	Functions	References
Natural	Folic acid	*S*-adenyl methionine generation	Park et al., 2012
	vitamins B6 and B12	*S*-adenyl methionine generation	Park et al., 2012
	Vitamin C	TET2 activator	DiTroia et al., 2019
	Methionine	Increase DNA methylation	Waterland, 2006
	Catechin, epicatechin	DNMTs inhibitor	Nagai et al., 2004
	EGCG	DNMTs inhibitor	Wong et al., 2011
	Resveratrol	DNMTs inhibitor	Aldawsari et al., 2016
	Quercetin	DNMTs inhibitor	Liu C. M. et al., 2015
Synthetic	Azacytidine	DNMTs inhibitor	Strand et al., 2020
	Decitabine	DNMTs inhibitor	Zhuang et al., 2017
	RG108	DNMTs inhibitor	Stenzig et al., 2018
	Hydralazine	DNMTs inhibitor	Kao et al., 2011
	GLP-1 agonists	DNMTs inhibitor and TET2 activator	Scisciola et al., 2020
	SGLT2 inhibitors	Not available	Marumo et al., 2015

*EGCG, epigallocatechin-3-O-gallate; decitabine, 5-Aza-2′-deoxycytidine; TET, 10–11 translocations; DNMT, DNA methyltransferases.*

Nutrition and dietary compounds are identified to regulate DNA methylation. Nutrients implicated in one-carbon metabolism, including folates (vitamin B9), vitamins B6 and B12, choline, and methionine, are involved in DNA methylation for their essential role in the generation of *S*-adenyl methionine, the methyl group donor for DNA methylation (Park et al., 2012). Folic acid and folate drugs are used as therapeutics. For instance, folic acid or vitamins B6 and B12 deficiencies can increase homocysteine levels, induce endothelial dysfunction, and aggravate atherosclerosis. On the contrary, dietary supplementation with folic acid can improve DNA methylation status, decrease inflammatory molecule levels, benefit atherosclerosis and reduce the risk of stroke (Wang et al., 2007; Hou and Zhao, 2021). Additionally, diet nutrition with folate and vitamin B6 has been recognized to improve memory and daily activities in AD patients (Chan et al., 2008). In addition, supplement methionine can increase DNA methylation (Waterland, 2006). Furthermore, vitamin C, as an antioxidant, can regulate the activity of TET and is involved in TET-mediated DNA methylation (DiTroia et al., 2019). Previous studies demonstrated that polyphenols [catechin, epicatechin, epigallocatechin-3-*O*-gallate (EGCG), and resveratrol] and bioflavonoids (quercetin, fisetin, and myricetin) inhibit DNA methylation by repressing DNMTs (Chistiakov et al., 2017). Catechin and epicatechin serve as DNMTs inhibitors through inhibiting human liver catechol-*O*-methyltransferase-mediated *O*-methylation of catechol estrogens (Nagai et al., 2004). EGCG is a major green tea polyphenol and can inhibit the activity of DNMTs such as DNMT1, DNMT3a, and DNMT3b (Wong et al., 2011). Besides, resveratrol has been recognized as DNMTs inhibitors to inhibit DNMTs expression (Aldawsari et al., 2016). Quercetin can protect against nickel-induced liver injury by suppressing DNMTs activity and decreasing the DNA methylation level of the NF-E2 related factor 2 (Liu C. M. et al., 2015). The ability of dietary nutrition offers promising therapies for vascular aging-related diseases by modulating DNMTs activities and DNA methylation.

DNA methyltransferases inhibitors are divided into two classes included nucleoside analogs inhibitors and non-nucleoside analogs inhibitors (Nicorescu et al., 2019). Nucleoside analogs inhibitors can incorporate into DNA during cell cycle and sequester DNMTs by regulating their proteasomal degradation. 5-azacytosine (azacytidine) and 5-Aza- 2′-deoxycytidine (5Aza, decitabine), DNA hypomethylation agents, may beneficial in CVD, cerebrovascular disease, and kidney diseases. Azacytidine serves as a DNMT1 inhibitor, upregulated the expression of PTEN, reduced inflammatory factors secretion, and inhibited platelet-derived growth factor stimulated SMCs de-differentiation (Strand et al., 2020). Besides, silencing DNMTs in ECs by 5Aza or *siRNA*, decreased the level of DNA methylation and attenuated ECs inflammation. Administration of 5Aza in mouse atherosclerosis models decreases the formation of atherosclerotic lesions and promotes the prognosis of atherosclerosis (Dunn et al., 2014). Administering 5Aza combined with specific task training helps recover chronic stroke [144]. Decitabine can ameliorate atherosclerotic lesion, inhibit DNMT1 activity, and downregulate global DNA methylation level (Zhuang et al., 2017). Non-nucleoside analogs such as RG108 and hydralazine are developed to overcome the non-specificity and cytotoxicity of the nucleoside inhibitors (Nicorescu et al., 2019). RG108 can inhibit DNMT1 activity by binding to its active site. Stenzig et al. (2018) indicated that RG108 significantly attenuated global DNA methylation in cardiomyocytes and is associated with decreased cardiac hypertrophy. Hydralazine, an anti-hypertensive drug, can repress DNMT1 and DNMT3a mRNA expression and activity by suppressing Erk signaling pathway (Deng et al., 2003). Hydralazine-mediated the promoter region of *SERCA2a* demethylation can improve cardiac function (Kao et al., 2011).

Diabetes is characterized by hyperglycemia and act as a significantly risk factor of cardiovascular and kidney disorders. Numerous lines of evidence supported that diabetes is linked with DNA methylation (Salameh et al., 2020). Therefore, DNA methylation-based therapies can benefit diabetes and reduce diabetes-related vascular complications. Cells treated with high glucose exhibited lower DNA methylation levels of *NF-*κ*B* and *SOD2*, while co-treatment with GLP-1 agonists reversed these effects by decreasing DNMT1 and DNMT3a mRNA and protein levels and increasing TET2 mRNA and protein levels (Scisciola et al., 2020). Additionally, it has been reported that *SGLT2* gene is hypomethylated in kidney proximal tubules, which suggest that SGLT2 inhibitors may be a DNA methylation modulators (Marumo et al., 2015).

Therefore, a better understanding of the mechanisms and roles of DNA methylation in the physiological and pathological process of vascular aging-related diseases might lead to the identification of promising biomarkers and therapeutic drugs. Much work is needed to validate the potential application of DNA methylation in vascular disorders.

## Perspectives and Conclusion

With the increase in the aging population, there is an urgent need to identify reliable and effective diagnostic tools and therapies for early diagnosis and treatment of vascular aging. Endothelial dysfunction, VSMCs proliferation and migration, macrophages polarization, and aberrant DNA methylation are implicated in the development and progression of vascular inflammation and vascular aging-related diseases. Notably, DNA methylation is involved in regulating the expression of genes in these mechanisms. In this review, we summarize the cellular and functional alterations in the vascular system during the aging process and concentrated on the roles and mechanisms of DNA methylation in vascular aging-related CVD, cerebrovascular diseases, and kidney diseases. DNA methylation plays a vital role in vascular aging progression and might be a potential biomarker for diagnosis and therapeutic target in treating vascular aging-related diseases. DNA methylation promotes a better understanding of the underlying mechanisms of vascular aging and related diseases and ultimately facilitates the development of effective interventions for vascular aging-related disorders. Regarding the future clinical prospect of DNA methylation, numerous biological explorations should be conducted to clarify the feasibility of DNA methylation in the diagnosis, treatment, and prognosis of vascular aging-related diseases.

## Author Contributions

HX collected the literature and wrote the manuscript. SL drew the figures and supervised the manuscript. Y-SL conceived the idea and had been involved in manuscript conception and drafting. All authors read and approved the final manuscript.

## Conflict of Interest

The authors declare that the research was conducted in the absence of any commercial or financial relationships that could be construed as a potential conflict of interest.

## Abbreviations

ECs: endothelial cells
VSMCs: vascular smooth muscle cells
CVD: cardiovascular disease
AD: Alzheimer’s disease
4mC: *N*^4^-methylcytosine
5mC: *N*^5^-methylcytosine
6mA: *N*^6^-methyladenine
DNMTs: DNA methyltransferases
TETs: 10–11 translocations
5hmC: 5-hydroxymethylcytosine
5fC: 5-formylcytosine
5caC: 5-carboxylcytosine
TDG: thymine DNA glycosylase
BER: base excision repair
ox-LDL: oxidized low-density lipoprotein
ROS: reactive oxygen species
LDL: low-density lipoprotein
eNOS: endothelial nitric oxide synthase
IL-1α: interleukin-1 α
5Aza: 5-aza-2 ′ -deoxycytidine
RAAS: rennin–angiotensin–aldosterone system
HF: heart failure
AMI: acute myocardial infarction
CHD: coronary heart disease
CKD: chronic kidney disease
EGCG: epigallocatechin-3-*O*-gallate.
